# Hereditary renal glycosuria, diabetes and responses to SGLT2 inhibitor

**DOI:** 10.1111/1753-0407.13254

**Published:** 2022-02-28

**Authors:** Qian Ren, Siqian Gong, Xuyao Han, Linong Ji

**Affiliations:** ^1^ Department of Endocrinology and Metabolism Peking University People’s Hospital Beijing China

**Keywords:** diabetes, genetic renal glycosuria, SGLT2 inhibitor, SLC5A2, 糖尿病, 遗传性肾性糖尿, SGLT2抑制剂, SLC5A2

## Abstract

**Aims:**

To present the clinical features of two rare cases with hereditary renal glycosuria and diabetes, explore their responses to sodium‐glucose cotransporter 2 (SGLT2) inhibitor, and summarize the reported solute carrier family 5 member 2 (SLC5A2) mutations and related phenotypes.

**Methods:**

Two patients were followed up for 6.5 and 3 years respectively. SLC5A2 and hepatocyte nuclear factor 1‐alpha (HNF1A) gene were sequenced. We used the flash glucose monitoring system to evaluate the efficacy of SGLT2 inhibitor treatment. Then we retrieved all the literature and analyzed SLC5A2 gene mutations and the phenotypes.

**Results:**

During long‐time follow up, the two patients had frequent unproportional renal glycosuria in the morning even when their fasting serum glucose was only slightly increased. A novel rare mutation V359G and a pathogenic rare mutation ivs7 + 5G > A in SLC5A2 gene were found respectively. In Case 1, the 24 h glucose excretion was 2.2 g/d and increased to 103 g/d after dapaglifozin treatment, whereas the average glucose (6.33 ± 1.56 vs. 6.28 ± 1.74 mmol/L), and time in range (TIR) (95% vs. 93%) were similar. In Case 2, the 24 h glycosuria was 121.4 g/d and increased to 185.8 g/day after dapaglifozin add‐on therapy, with a further reduction of average glucose (9.11 ± 2.63 vs. 7.54 ± 2.39 mmol/L, *p* < 0.001) and better TIR (70% vs. 84%). We reviewed 139 cases with hereditary renal glycosuria and SLC5A2 gene mutation. The urine glucose was highest in patients with homozygous mutations [64.0(36.6–89.6)g/24 h] compared with compound heterozygous mutations [25.9(14.4–41.2)g/24 h] and heterozygous mutations [3.45(1.41–7.50)g/24 h] (*p* < 0.001).

**Conclusions:**

Genetic renal glycosuria could not protect individuals completely from developing diabetes. Patients with SGLT2 gene mutations are still responsive to the SGLT2 inhibitor treatment.

## Graphical abstract

**Highlights**

- Genetic renal glycosuria could not protect individuals completely from developing diabetes.
- However, Patients with SGLT2 gene mutations are still responsive to the SGLT2 inhibitor treatment.

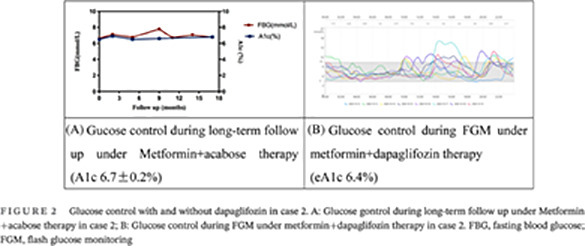

## 摘要

**目的:**

介绍2例罕见的遗传性肾性糖尿和糖尿病患者的临床特征，探讨其对SGLT2抑制剂的反应，并总结已报道的SLC5A2突变和相关表型。

**方法:**

对2例患者分别随访6.5年和3年。对SLC5A2和HNF1A基因进行测序。我们使用快速血糖监测系统来评估SGLT2抑制剂治疗的疗效。然后检索所有文献，分析SLC5A2基因突变和表型。

**结果:**

在长期随访中，2例患者在空腹血糖仅轻微升高的情况下，早晨仍有频繁的肾性糖尿。SLC5A2基因中分别发现一个新的罕见突变V359G和一个致病性罕见突变ivs7+5G>A。在病例1中，24小时葡萄糖排泄量为2.2g/d，并且在达格列净治疗后增加到103g/d，而平均血糖(6.33±1.56 vs 6.28±1.74mol/L)和范围内时间(TIR) (95% vs 93%)(在治疗前后)相似。在病例2中，加用达格列净治疗后24h尿糖从121.4克/天，增加至185.8克/天，平均血糖进一步降低(9.11±2.63 vs 7.54±2.39mol/L，P<0.001)，TIR更好(70% vs 84%)。我们回顾了139例遗传性肾性糖尿和SLC5A2基因突变的病例。与复合杂合子突变[25.9(14.4‐41.2)g/24h]和杂合子突变[3.45(1.41‐7.50)g/24h]相比，纯合子突变[64.0(36.6‐89.6)g/24h]患者的尿糖最高(P<0.001)。

**结论:**

遗传性肾性糖尿不能完全保护个体不患糖尿病。SGLT2基因突变的患者对SGLT2抑制剂治疗仍有反应。

## 1 INTRODUCTION

Glucose‐lowering therapies has changed greatly in patients with type 2 diabetes mellitus (T2DM) in recent years. Sodium glucose cotransporter 2 (SGLT2) inhibitors that can lower the A1c level about 1%–2% in patients with T2DM^2^ had already comprised nearly one fifth of new prescriptions by 2017 in the United States.^1^ SGLT2 was mostly expressed on renal tubules and is coded by the solute carrier family 5 member 2 (SLC5A2) gene. Interestingly, some people with familial renal glycosuria (FRG) were found carrying SLC5A2 and hepatocyte nuclear factor 1‐alpha (HNF1A) gene mutations. Their clinical feature was a natural replication of SGLT2 inhibition in type 2 diabetic patients taking SGLT2 inhibitors.^3^ Theoretically, the SLC5A2 gene mutation carriers might have lower risk for developing diabetes.

Here, we report two diabetic patients with hereditary renal glycosuria caused by SLC5A2 gene mutations. In this short communication, we describe their clinical features and their responses to SGLT2 inhibitor treatment. In addition, we summarize SLC5A2 gene mutations and the related phenotypes by reviewing all the relevant publications between 2002 and 2020.

## 2 METHODS

### 2.1 Patients

We identified two patients with early‐onset diabetes who had been referred for genetic testing by clinicians. Both of them were treated with oral antidiabetic drugs and had satisfactory glucose control. We followed one patient for 6.5 years and another for 3 years, and they both had frequent unproportional renal glycosuria when their fasting serum glucose was only slightly increased above the normal range.

### 2.2 Genetic analysis

Informed consent was approved by the Ethics Committee of Peking University People’s Hospital and obtained from the two patients. First, we measured the total urine glucose excretion during 24 h in the two patients. Sequencing of SLC5A2 gene and HNF1A gene were performed on DNA from two patients. Other etiologies of renal glycosuria such as Fanconi syndrome were excluded by clinical evaluations.

### 2.3 Pathogenicity prediction

The function of mutations was predicted according to American College of Medical Genetics (ACMG) recommendations.^4^ First, we evaluated the mutation frequencies in mBiobank (ChinaMAP), which was a deep whole genome sequences database of 10 588 Chinese individuals.^5^ Then online in silico tools were used, including SIFT (http://sift.jcvi.org) and Polyphen 2 (http://genetics.bwh.harvard.edu/pph2/).

### 2.4 Review of reported SLC5A2 mutations and quantity of urine glucose

We searched PubMed from 1st January 2002 to June 30th 2021. The key words of our search included genetic renal glycosuria, SLC5A2, SGLT2, and familial renal glucosuria. All the studies included were case reports, clinicogenetic study and retrospective study of patients with renal glucosuria. We analyzed SLC5A2 gene mutations and the related phenotypes, focusing on the quantity of urine glucose. We use the normal range of urine glucose excretion 0.13–0.32 g/1.73 m^2^ per day as the reference.^6^

### 2.5 Responses to SGLT2 inhibitors

In order to further evaluate the two patients’ responses to SGLT2 inhibitors, we used the flash glucose monitoring (FGM) system. Each patient wore an FGM sensor for 14 days blindly and the glucose data from the sensor were downloaded for analysis after 14 days. 24 h urine was collected on the seventh day of dapaglifozin treatment for quantification of 24 h urine glucose excretion.

During FGM, for Case 1, metformin (1.5 g/d) was treated in the first 7 days before switched to dapaglifozin (10 mg/d) in the last 7 days of FGM. For Case 2, who had already been treated with metformin (1.5 g/d) and dapaglifozin (10 mg/d) for 1.5 years, this treatment modality was kept during the first 7 days, and during the last 7 days of FGM, dapaglifozin was temporarily discontinued.

## 3 RESULTS

### 3.1 Clinical observation

#### 3.1.1 Case 1

**FIGURE 1:**
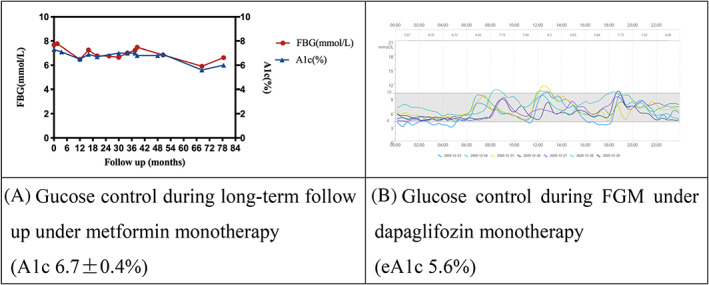
Glucose control with and without dapaglifozin in case 1. A: Glucose control during long‐term follow up under metformin monotherapy in case 1; B: Glucose control during FGM under dapaglifozin monotherapy in case 1. FBG, fasting blood glucose; FGM, flash glucose monitoring

Male, 45 years old, body mass index (BMI) 26.4 kg/m^2^, diagnosed with T2DM 11 years ago. He was treated with metformin mono therapy 1.5 g/d. During 6.5 years’ regular follow up in our clinic, his A1c was 6.7 ± 0.4% (50 ± 4.4 mmol/mol), with highest and lowest value of 5.6% (38 mmol/mol) and 7.3% (56 mmol/mol) as shown in Figure 1A. His average systolic blood pressure (SBP) was 121 ± 9 mm Hg, and diastolic blood pressure (DBP) was 83 ± 2 mm Hg during follow‐up. Carotid and lower extremity artery ultrasounds were normal. He had normal fundus, urine albumin creatine ratio (UACR) was 2.45 (0.60–3.35) mg/g, and estimated glomerular filtration rate (eGFR) was 111.6 ± 4.6 ml/min*1.73m^2^. He had frequent unproportional renal glycosuria (5.5–28 mmol/L) in the morning even when his fasting serum glucose was only slightly increased above the normal range (6.96 ± 0.52 mmol/L, lowest and highest values were 5.91 and 7.77 mmol/L) during metformin mono therapy.

#### 3.1.2 Case 2

**FIGURE 2:**
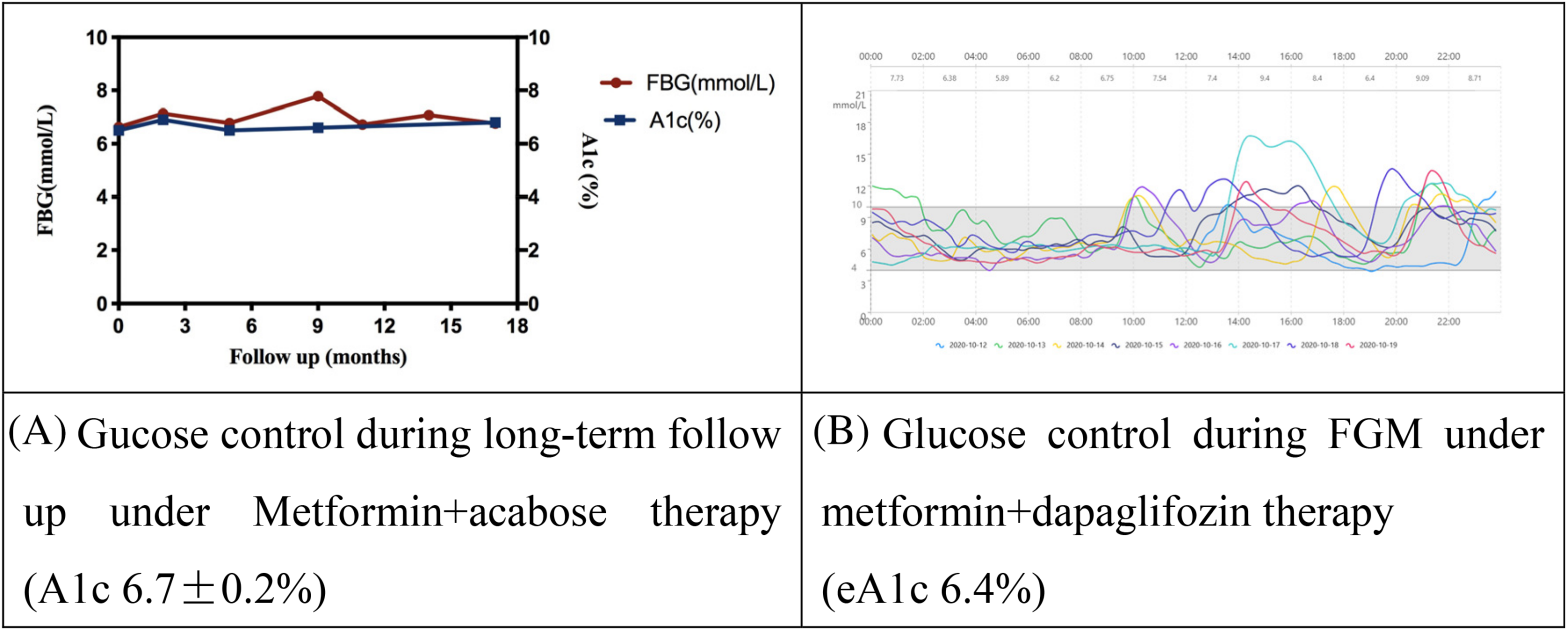
Glucose control with and without dapaglifozin in case 2. A: Glucose gontrol during long‐term follow up under Metformin+acabose therapy in case 2; B: Glucose control during FGM under metformin+dapaglifozin therapy in case 2. FBG, fasting blood glucose; FGM, flash glucose monitoring

Male, 37 years old, BMI 26.9 kg/m^2^, diagnosed with type 2 diabetes 4 years ago in routine physical examination. He was treated with metformin 1.5 g/d combined with acarbose 150 mg/d and had been followed up in our clinic for 3 years. During the first 1.5 years’ follow‐up, his A1c was 6.7 ± 0.2% (50 ± 2.2 mmol/mol), highest and lowest values were 6.9% (52 mmol/mol) and 6.5% (48 mmol/mol), respectively as shown in Figure 2A. His average SBP was 113 ± 6 mm Hg, and DBP was 74 ± 7 mm Hg during follow‐up. He had normal fundus, UACR was 62.4 ± 16.6 mg/g, and eGFR 121.3 ± 3.5 ml/min*1.73m^2^. He was also found to have unproportional renal glycosuria (14‐ > 55 mmol/L) in the morning when his fasting serum glucose was only slightly increased (6.98 ± 0.40 mmol/L, lowest and highest values were 6.63 and 7.78 mmol/L) during metformin plus acarbose therapy.

### 3.2 Genetic testing results

**FIGURE 3:**
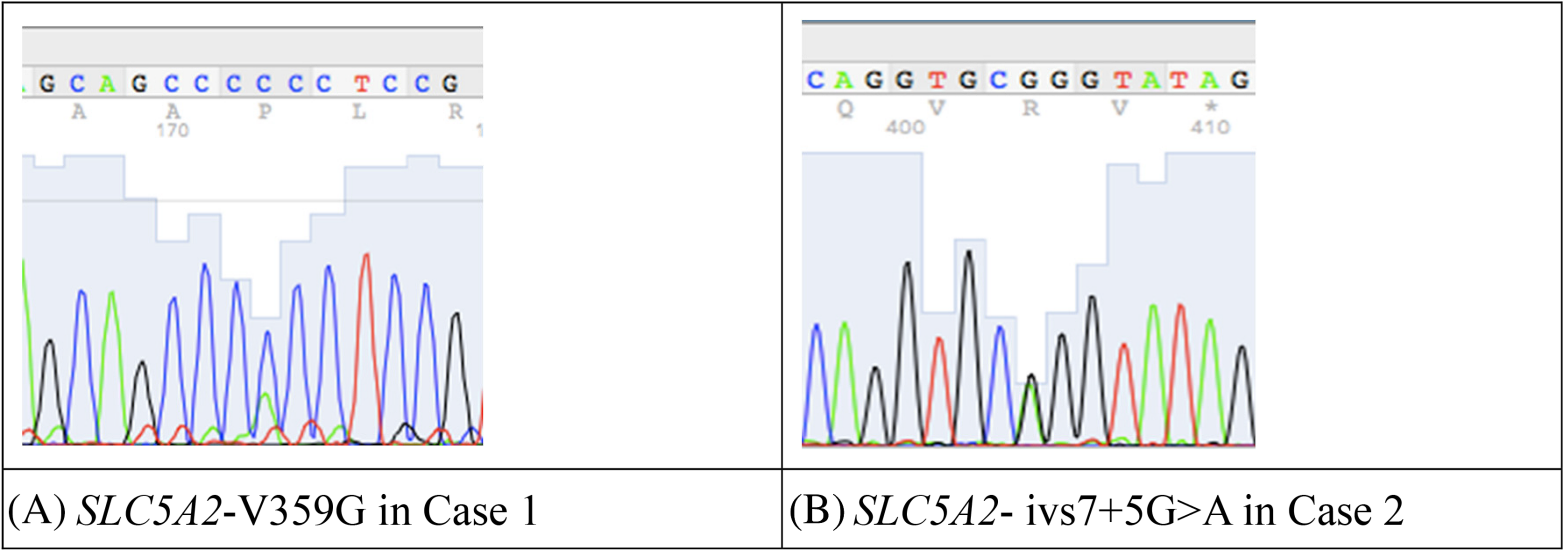
The SLC5A2 gene mutations in both cases. SLC5A1, solute carrier family 5 member 2. A: SLC5A2‐V359G in Case 1; B: SLC5A2‐ivs7+5G>A in Case 2

We found that Case 1 had a novel rare mutation V359G in the SLC5A2 gene (Figure 3A), which was not found in mBiobank (ChinaMAP) and predicted to be pathogenic according to ACMG recommendations.^4^ Case 2 had pathogenic rare mutation ivs7 + 5G > A in the SLC5A2 gene (Figure 3B), which has been reported as a hot spot mutation causing hereditary renal glycosuria.^7^

### 3.3 Responses to SGLT2 inhibitor treatment

In Case 1, during the first 7 days on metformin and second 7 days on dapaglifozin (Figure 1B), time in range (TIR) was 95% versus 93%, the average glucose was 6.33 ± 1.56 versus 6.28 ± 1.74 mmol/L (*p* = 0.663), coefficient of variation (CV) was 24.7 versus 27.7%, mean amplitude of glycemic excursion (MAGE) was 3.27 versus 4.50 mmol/L, and mean of daily differences (MODD) was 1.27 versus 1.27 mmol/L respectively. The 24 h urine glucose excretion was 2.2 g/d (normal range 0.13–0.32 g/1.73m^2^/24 h) with metformin treatment, and the fasting self‐monitoring of blood glucose (SMBG) was 5.7 and 5.5 mmol/L on urine sample collection day and the next day. The 24 h urine glucose excretion with dapaglifozin treatment was 103 g/day.

In Case 2, during recent 1.5 years’ follow‐up, he was treated with metformin 1.5 g/d plus dapagliflozin 10 mg/d because of persistent microalbuminria. He was not treated with angiotensin‐converting enzyme inhibitor (ACEi) or angiotensin‐II‐receptor blocker (ARB) before starting dapagliflozin because he did not have hypertension, and the indications of ACEi and ARB in China are hypertension or diabetic kidney disease combined with hypertension. After dapagliflozin add‐on therapy, his average UACR was 45.7 ± 10.7 mg/g, eGFR 120.9 ± 5.0 ml/min*1.73m^2^, and A1c 6.1 ± 0.1% (43 ± 1.1 mmol/mol), compared with A1c 6.7 ± 0.2% (50 ± 2.2 mmol/mol) before dapagliflozin add‐on therapy. During FGM, under 7‐day treatment of metformin+dapaglifozin (Figure 2B) and 7‐day treatment of metformin mono therapy, TIR was 84% versus 70%, the average glucose was 7.54 ± 2.39 versus 9.11 ± 2.63 mmol/L (*p* < 0.001), CV was 31.7 versus 28.8%, MAGE was 5.60 versus 5.71 mmol/L, and MODD was 2.25 versus 2.61 mmol/L, respectively. The 24 h urine glucose excretion with metformin plus dapagliflozin treatment was 185.8 g/day. The 24 h urine glucose excretion was 121.4 g/d after discontinuation of dapagliflozin, and the fasting SMBG was 7.5 and 7.4 mmol/L on urine sample collection day and the next day.

### 3.4 Literature review of reported SLC5A2 mutations and quantity of urine glucose

A total of 139 cases were reported in 21 publications as shown in Supplementary Table A1, including three types of mutations: homozygous (28 cases, 20.3%), compound heterozygous (33 cases, 23.9%), and heterozygous (76 cases, 55.1%). The quantity of urine glucose was significantly highest in patients with homozygous mutation (64.0[36.6–89.6]g/24 h) compared with compound heterozygous (25.9[14.4–41.2]g/24 h) and heterozygous (3.45[1.41–7.50]g/24 h) (*p* < 0.001). Only two patients had diabetes (1.4%), and both of them carried heterozygous mutation. There were 17 patients carrying the same mutation of ivs7 + 5G > A as in Case 2. The urine glucose excretion varied between 0.75 and 62.3 g/24 h.

## 4 DISCUSSION

Here, we reported long‐term follow‐up of two diabetic cases with hereditary renal glycosuria caused by SLC5A2 mutations and they were still responsive to SGLT2 inhibitor treatment.

The two patients had common clinical features of persistent renal glycosuria concomitant with optimal control of diabetes during long‐term follow‐up, which implied that they had a reduction in the renal glucose threshold. Further genetic analysis found that their glycosuria was caused by the SLC5A2 gene mutations. Previous studies reported that SLC5A2 gene mutations were responsible for most of familial renal glycosuria (FRG) cases, and at least 89 SLC5A2 gene mutations from 138 cases were reported. It is noteworthy that among those FRG cases, only four cases (2.9%) had diabetes, suggesting that SLC5A2 gene mutation might be protective to diabetes.^8^

The second interesting finding of this case study was that, in the patient with the SLC5A2 gene mutation, who already had inherited defects of SGLT2, SGLT2 inhibitor was still effective. In previous studies, the glycosuria in FRG patients could range from less than 1 g to 202 g/1.73m^2.^ ^3^ In this case, it seemed that the mutations did not totally abolish the activity of glucose transporter in the renal proximal tubule, which could still respond to SGLT2 inhibitor. Further study is needed to explore the influence of FRG’s genetic defect on SGLT2 inhibitor’s binding site. SGLT2 inhibitor is a novel therapeutic strategy in improving hyperglycemia and also renal protection in patients with type 2 diabetes.^2^ Our observation suggests that, even in diabetic patients with decreased renal glucose threshold, SGLT2 inhibitors could also be effective in blood glucose control. The effectiveness of SGLT2 inhibitors might depend on the amount of urine glucose excretion at baseline rather than the SLC5A2 gene mutation itself.

## AUTHOR CONTRIBUTIONS

Qian Ren and Siqian Gong drafted the manuscript. Xuyao Han interpreted the laboratory results. Xuyao Han and Linong Ji critically reviewed the manuscript. Linong Ji is the guarantor of this work, and he had full access to all the data in the study and takes responsibility for the integrity of the data and the accuracy of the data analysis.

## DISCLOSURE

There is no potential conflict of interest associated with this manuscript.

## Supporting information


**Supplementary Table A1.** Reported SLC5A2 mutations and quantity of urine glucose

## Data Availability

The data are available from the corresponding author on reasonable request.
